# Influence of Electroporation Medium on Delivery of Cell-Impermeable Small Molecules by Electrical Short-Circuiting via an Aqueous Droplet in Dielectric Oil: A Comparison of Different Fluorescent Tracers

**DOI:** 10.3390/s22072494

**Published:** 2022-03-24

**Authors:** Yuki Watanabe, Hirohito Nihonyanagi, Rika Numano, Takayuki Shibata, Kazunori Takashima, Hirofumi Kurita

**Affiliations:** 1 Department of Applied Chemistry and Life Science, Toyohashi University of Technology, Toyohashi 441-8580, Aichi, Japan; watanabe.yuki.mn@tut.jp (Y.W.); nihonyanagi.hirohito.mt@tut.jp (H.N.); numano@tut.jp (R.N.); takashima@chem.tut.ac.jp (K.T.); 2 The Electronics-Inspired Interdisciplinary Research Institute (EIIRIS), Toyohashi University of Technology, Toyohashi 441-8580, Aichi, Japan; 3 Department of Mechanical Engineering, Toyohashi University of Technology, Toyohashi 441-8580, Aichi, Japan; shibata@me.tut.ac.jp

**Keywords:** electroporation, membrane permeabilization, electric conductivity, endocytosis

## Abstract

Membrane permeabilization stimulated by high-voltage electric pulses has been used to deliver cell-impermeable exogenous molecules. The electric field effect on the cells depends on various experimental parameters, such as electric field strength, the number of electric pulses, and the electroporation medium. In this study, we show the influence of the electroporation medium on membrane permeabilization stimulated by electrical short-circuiting via an aqueous droplet in dielectric oil, a novel methodology developed by our previous investigations. We investigated the membrane permeabilization by three methods, influx of calcium ions, uptake of nucleic acid-binding fluorophores (YO-PRO-1), and calcein leakage. We demonstrated that the external medium conductivity had a significant impact on the cells in all described experiments. The short-circuiting using a low-conductivity electroporation medium enhanced the formation of both transient and irreversible membrane pores. We also found that clathrin-mediated endocytosis contributed to YO-PRO-1 uptake when a cell culture medium was used as an electroporation medium.

## 1. Introduction

Electroporation has been widely used for delivering various cell-impermeable molecules into cells both in vitro and in vivo [1,2,3,4,5,6,7]. As its name suggests, when an intense external electric pulse is applied to cells, it eventually increases membrane permeability due to the formation of transient membrane pores. This phenomenon is the crucial process for delivering the extracellular molecules. Nucleic acids have been the most popular molecules to be delivered into cells; for example, gene transfection is used for genome editing [8,9,10,11,12,13,14,15], generation of induced pluripotent stem (iPS) cells [16,17,18,19], and gene therapy [20,21,22]. Electroporation has also allowed for the delivery of small molecules, such as antitumor drugs. This application is known as electrochemotherapy (ECT) [3,23,24].

Various analyses have investigated transient membrane pore formation [4,25,26]. Electroporation and its extent are most often determined by detecting the increased transport of impermeant molecules across the membrane. For example, uptake of nucleic acid-binding fluorophores, such as propidium iodide (PI), ethidium bromide (EtBr), and YO-PRO-1 iodide, is the most common way to investigate electroporation. These fluorophores are impermeant molecules that enter cells through an electroporated membrane. After entering the cells, they bind to nucleic acids and show significant fluorescence enhancement. The influx of ions presenting in the cytoplasm at low concentrations, such as calcium ions, can also indicate electroporation [27]. In this case, a fluorescent ion indicator with cell-permeable acetoxymethyl (AM) ester form (e.g., Fluo-4 AM) can enter passively into cells. They are cleaved in the cytosol by intracellular esterases to impermeant dyes. When the cells loaded with the indicator are electroporated with the interest ions, fluorescence increase can be observed using a fluorescence microscope or flow cytometry. Monitoring the extracellular release of membrane-impermeable intracellular molecules can detect electroporation [28,29,30]. The efflux of cell-impermeable small molecules can be analyzed by appropriate methods. Calcein acetoxymethyl ester (calcein-AM), a non-fluorescent cell-permeable compound, can be used to monitor the efflux of intracellular molecules. Intracellular esterases hydrolyze calcein-AM to produce calcein (MW = 623), an impermeant fluorescent dye. A decrease in the fluorescence intensity of the cells indicates the leakage of intracellular small molecules.

We have reported novel gene electrotransfer using an electrical short-circuiting via a cell suspension droplet in dielectric oil [31,32,33]. An intense DC electric field can induce droplet deformation between an electrode pair, resulting in instantaneous short-circuiting caused by bridging two electrodes. We have demonstrated successful gene transfection into various mammalian cells, including bovine and swine fibroblast cells that are difficult to be transfected by lipofection [34]. The previous investigation has also shown that droplet-based electroporation allows for delivery of proteins into animal sperm. This methodology has several features over conventional electroporation. First, electroporation can be performed with a DC high-voltage power supply; therefore, a comparatively expensive electric pulse generator is not required. Second, the droplet system is suitable for miniaturization using microfluidics. We have also elucidated the exogenous gene expression mechanism in mammalian cells [35]. The previous investigation demonstrated the following highlights: (1) the electrical stimulation increases transient membrane permeability to calcium ions and YO-PRO-1 fluorescent dyes, indicating transient membrane pore formation. (2) The short-circuiting via an aqueous droplet stimulated endocytosis, contributing to successful exogenous gene expression.

In this paper, we performed flow cytometric assays to investigate transient membrane permeabilization. Although membrane permeabilization stimulated by short-circuiting was already demonstrated in our previous studies, the influence of experimental parameters was not well-elucidated. Among the various experimental parameters, we focused on an electroporation medium. We prepared electroporation media with different ion strengths (i.e., electric conductivity). The influence of the ion strength on membrane pore formation was investigated by different methods, influx of calcium ions, YO-PRO-1 uptake, and calcein leakage. In the previous study, the contribution of endocytosis to the transportation of small molecules remained to be elucidated. Therefore, we demonstrated the effect of endocytosis inhibition on YO-PRO-1 uptake.

## 2. Materials and Methods

### 2.1. Cell Culture

The experiments were performed using Jurkat cells (RIKEN BioResource Research Center, Tsukuba, Japan). Jurkat cells were maintained in RPMI-1640 with l-glutamine and phenol red (FUJIFILM Wako Pure Chemical, Osaka, Japan), 10% fetal bovine serum (FBS, Thermo Fisher Scientific, Waltham, MA, USA), 100 units/mL penicillin, and 100 μg/mL streptomycin (PS, FUJIFILM Wako Pure Chemical) at 37 °C, 5% CO_2_. Cells were passed every 2–3 days and a day before the experiment.

### 2.2. Electroporation

**Figure 1:**
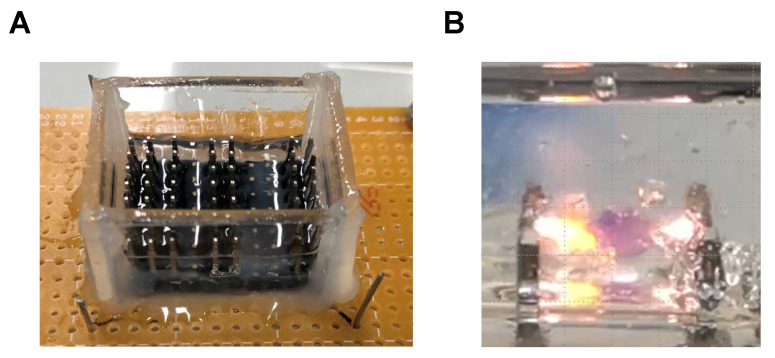
Experimental apparatus for an aqueous droplet actuation in dielectric oil [35]. (**A**) Overview of the apparatus. (**B**) Typical short-circuiting via an aqueous droplet by applying DC HV electric field. A 3.0 μL aliquot of RPMI-1640 was added to the silicone oil and 3.0 kV of DC HV was applied to the electrode with 5.08 mm gap.

Figure 1 shows the experimental setup. The apparatus was fabricated with pin headers and a printed circuit board as described in the previous paper [35]. The electrodes’ gap was 5.08 mm. The apparatus was filled with 1.5 mL fluorocarbon oil (Fluorinert, 3M, Tokyo, Japan), and then 1 mL silicone oil (KF96-100CS, 100 cSt kinematic viscosity, 2.74 relative dielectric constant, 965 kg/m^3^ density, Shin-Etsu Chemical, Tokyo, Japan) was added.

Before electroporation, Jurkat cells were harvested by centrifugation (10 min, 200× *g*, 4 °C) and resuspended in an appropriate electroporation medium. The cell concentration was determined using a Coulter counter (Z2, Beckman Coulter, Brea, CA, USA). The cell suspension (3.0 μL) containing 1.0 × 10^5^ Jurkat cells was dispensed in the silicone oil, and a high voltage was supplied using a DC high-voltage (HV) power supply (HAR-30R10, Matsusada Precision, Kusatsu, Japan). When the applied voltage was set to 3.0 kV, instantaneous short-circuiting could be induced. After short-circuiting, with a distinctive sound, the DC HV power supply was manually turned off.

### 2.3. Electroporation Media

To investigate the influence of electrical conductivity on transient membrane permeabilization, HEPES-buffered saline solution (HEPES-saline, 10 mM HEPES, 1 mM MgCl_2_, 140 mM NaCl, pH 7.4) and HEPES-buffered sucrose solution (HEPES-sucrose, 10 mM HEPES, 1 mM MgCl_2_, 250 mM sucrose, pH 7.4) were used for electroporation. The electrical conductivities of the media measured using a conductivity meter (LAQUA, Horiba, Kyoto, Japan) were 1.47 S/m and 0.06 S/m, respectively. RPMI-1640 was also used in YO-PRO-1 uptake assay and measurement of the loss of intracellular molecules. The electric conductivity was 1.34 S/m.

### 2.4. Measurement of Intracellular Calcium Ion

The influx of calcium ions presenting in the cytoplasm at a low concentration was measured to demonstrate transient membrane permeabilization stimulated by short-circuiting. The measurement of intracellular calcium ions was conducted as previously described [35], with some modifications: 4.0 × 10^6^ Jurkat cells suspended in Dulbecco’s phosphate-buffered saline without magnesium chloride and calcium chloride (D-PBS (-), FUJIFILM Wako Pure Chemical) were incubated with 4.5 μM Fluo-4 AM (Thermo Fisher Scientific) for 60 min at 37 °C. The Fluo-4-loaded cells were harvested by centrifugation and resuspended in HEPES-saline or HEPES-sucrose with and without 10 mM CaCl_2_. A 3.0 μL cell suspension containing 1.0 × 10^5^ cells was dispensed in the silicone oil, and a DC HV electric field was applied. Following the short-circuiting, the cells were immediately transferred to the same electroporation media without CaCl_2_, then incubated for 20 min at 37 °C. Since excessive calcium entry into cells leads to rapid cell death, cell viability was determined. Following the incubation for 20 min, 7-amino-actinomycin D (7-AAD, Beckman Coulter) was added to the cell suspension to stain the dead cells. The 7-AAD dye that is membrane impermeant and fluorescent when it binds to double-stranded DNA was used in place of PI to evaluate cell viability by flow cytometry. Following incubation with 7-AAD for 20 min, the fluorescence intensity of the cells was measured using flow cytometry.

### 2.5. YO-PRO-1 Uptake Assay

Uptake of cell-impermeable nucleic acid-binding fluorophores YO-PRO-1 iodide can also indicate electroporation. The measurement of YO-PRO-1 uptake was conducted to demonstrate transient membrane permeabilization stimulated by short-circuiting as previously described [35], with some modifications. A 3.0 μL cell suspension containing 1.0 × 10^5^ Jurkat cells and 1 μM YO-PRO-1 (Thermo Fisher Scientific) prepared with an appropriate electroporation medium was dispensed into the silicone oil, and the cells were treated with short-circuiting. After recovery of the cells, the cells were incubated in the same electroporation medium without YO-PRO-1 for 40 min at 37 °C. Following incubation with 7-AAD for 20 min, the fluorescence intensity of the cells was measured using flow cytometry.

### 2.6. Measurement of the Loss of Intracellular Molecules

The changes in the intracellular calcium ion concentration and YO-PRO-1 fluorescence intensity of the Jurkat cells demonstrated the influx of extracellular ions and molecules. Transient membrane permeabilization could stimulate the efflux of intracellular small molecules; therefore, the leakage of intracellular small molecules was monitored using calcein. Jurkat cells were preloaded with a fluorescent marker of cytosol, calcein-AM (Dojindo, Mashiki, Japan). Then, 4.0 × 10^6^ Jurkat cells were incubated with 0.5 μM calcein-AM in D-PBS (-) for 30 min at 37 °C. After incubation, the cells were harvested by centrifugation and resuspended in an appropriate electroporation medium, and they were used immediately in electroporation experiments. Following short-circuiting, the droplet was recovered and transferred to the same electroporation medium. The fluorescence intensity of the cells was immediately measured using flow cytometry.

### 2.7. The Effect of Endocytosis Inhibition on YO-PRO-1 Uptake

Endocytic pathways were considered to be different YO-PRO-1 uptake pathways from membrane permeabilization; therefore, the effect of endocytosis inhibition on YO-PRO-1 uptake was investigated. 4.0 × 10^6^ Jurkat cells in D-PBS (-) were treated with endocytosis inhibitor Pitstop 2-100 (Abcam, Cambridge, UK) for 30 min at 37 °C, 5% CO_2_ [35,36]. Following the Pitstop 2-100 treatment, the cell suspension was centrifuged and the cells were resuspended in RPMI-1640 without FBS. After addition of YO-PRO-1 (1 μM final concentration), short-circuiting using RPMI-1640 was performed, then YO-PRO-1 uptake and cell viability were measured (see Section 2.5). Since almost all endocytic pathways are metabolic energy-dependent processes, the effect of incubation temperature after short-circuiting on YO-PRO-1 uptake was investigated. After short-circuiting using RPMI-1640, cells were recovered into a microcentrifuge tube and incubated for 40 min at 4 °C or 37 °C.

### 2.8. Flow Cytometry

Flow cytometric analysis of individual cells was performed with a CytoFLEX flow cytometer (Beckman Coulter). The CytExpert software (Beckman Coulter) was used for data acquisition. The sample was filtered using a nylon cell strainer with 35 μm mesh size (Corning, NY, USA) before flow cytometry. At least 10,000 events were recorded for each experimental point. The Kaluza Analysis 2.1 software (Beckman Coulter) was used for the data analysis.

## 3. Results

### 3.1. Measurement of Intracellular Calcium Ion

**Figure 2:**
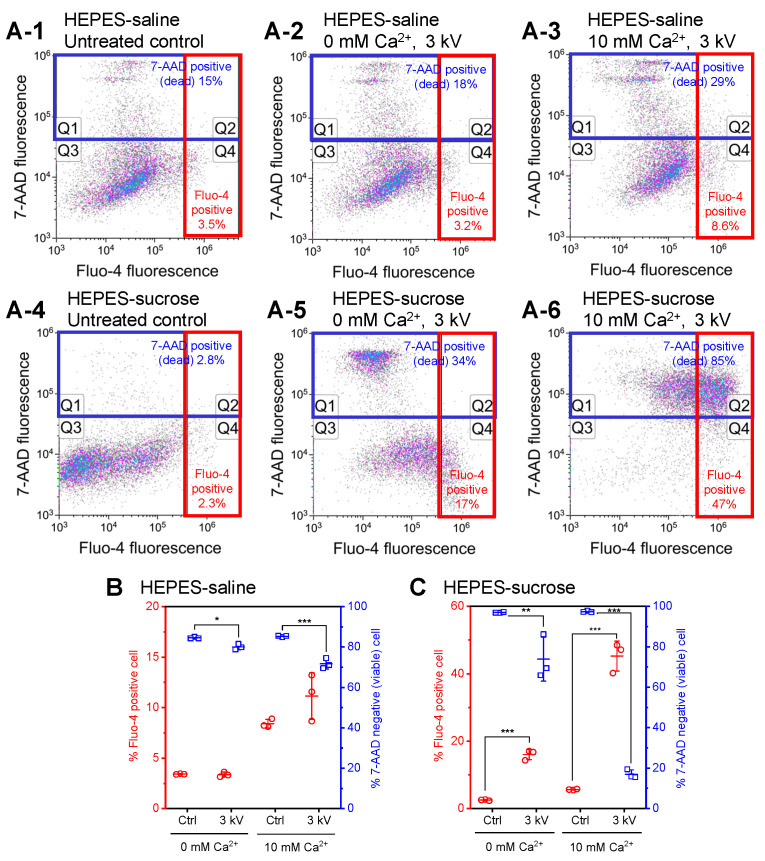

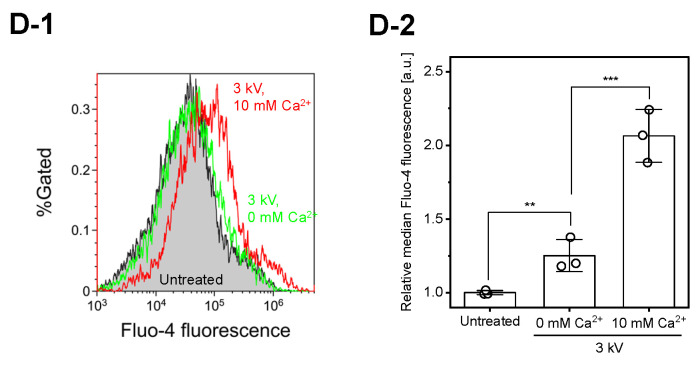
Measurement of intracellular calcium ion. (**A**) Intensity of fluorescence of Fluo-4 and 7-AAD was measured and plotted using a log scale. The percentages of 7-AAD-positive (dead) cells (Q1 + Q2) and Fluo-4-positive cells (Q2 + Q4) are shown. (**B**,**C**) The population of Fluo-4-positive cells and cell viability after short-circuiting using HEPES-saline (**B**) or HEPES-sucrose (**C**), as determined using flow cytometry. Data are expressed as the mean ± standard deviation (SD) of three independent measurements. Statistical significance was determined using one-way ANOVA followed by Tukey’s multiple comparison tests, * p<0.05, ** p<0.01, and *** p<0.001. (**D**) Changes in Fluo-4 fluorescence intensity after short-circuiting using HEPES-saline. Representative flow cytometry histograms (**D-1**) and relative median Fluo-4 fluorescence intensity (**D-2**) are shown. Data are expressed as the mean ± SD of triplicate measurements. Statistical significance was determined using one-way ANOVA followed by Tukey’s multiple comparison tests, ** p<0.01 and *** p<0.001.

**Figure 3:**
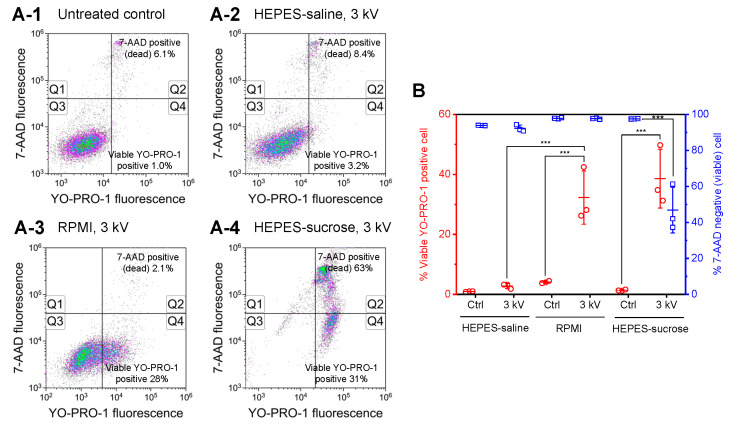
Uptake of YO-PRO-1 molecules measured by flow cytometry. (**A**) Intensity of fluorescence of YO-PRO-1 and 7-AAD was measured and plotted using a log scale. The percentages of 7-AAD-positive (dead) cells (Q1 + Q2) and YO-PRO-1-positive viable cells (Q4) are shown. (**B**) The population of YO-PRO-1-positive viable cells and cell viability after short-circuiting using different electroporation media, as determined using flow cytometry. Data are expressed as the mean ± SD of three independent measurements. Statistical significance was determined using one-way ANOVA followed by Tukey’s multiple comparison tests, *** p<0.001.

**Figure 4:**
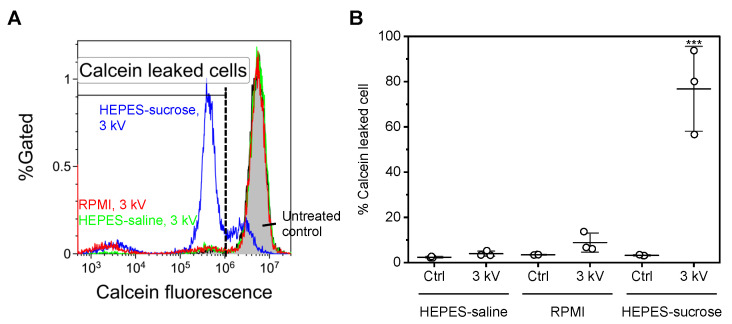
Measurement of the loss of intracellular molecules. (**A**) Representative flow cytometry histograms. The fluorescence intensity was determined for calcein. The dashed line indicates the threshold of the calcein-leaked cells. (**B**) The population of calcein-leaked cells after short-circuiting using different electroporation media, as determined using flow cytometry. Data are expressed as the mean ± SD of three independent measurements. Statistical significance was determined using one-way ANOVA followed by Tukey’s multiple comparison tests, *** p<0.001 vs. all the other conditions.

**Figure 5:**
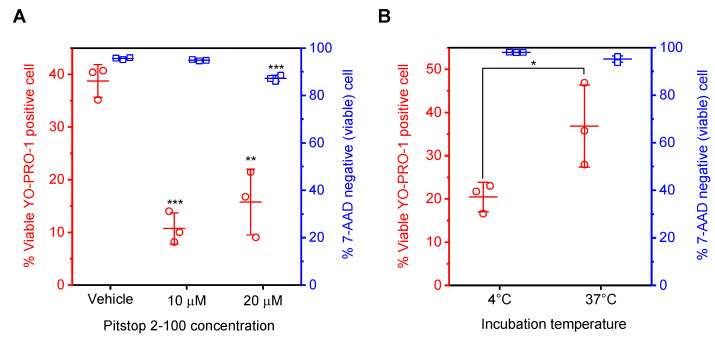
The effect of endocytosis inhibition on YO-PRO-1 uptake. (**A**) The effect of pretreatment of cells with an endocytosis inhibitor on YO-PRO-1 uptake. The population of YO-PRO-1-positive viable cells and cell viability after short-circuiting is shown. Data are expressed as the mean ± SD of triplicate measurements. Statistical significance was determined using one-way ANOVA followed by Tukey’s multiple comparison tests, ** p<0.01 and *** p<0.001 vs. vehicle. (**B**) The effect of incubation temperature after short-circuiting on YO-PRO-1 uptake. Data are expressed as the mean ± SD of triplicate measurements. Statistical significance was determined using Student’s *t*-tests, * p<0.05.

Figure 2 shows the results of intracellular calcium ion measurement. Flow cytometry was performed after incubation with 7-AAD. Representative flow cytometry density plots are displayed in Figure 2A. As shown in Figure 2(A-2,A-3), regardless of the calcium ion concentration in HEPES-saline, short-circuiting increased Fluo-4 fluorescence intensity in the density plots relative to the control shown in Figure 2(A-1). Figure 2(A-5) showed that short-circuiting using HEPES-sucrose buffer without calcium ion remarkably increased in the population of Fluo-4-positive cells and dead cells relative to the control experiment shown in Figure 2(A-4). The highest population of Fluo-4-positive cells was observed when using HEPES-sucrose buffer containing 10 mM Ca^2+^; however, most cells were dead cells (Figure 2(A-6)).

Figure 2B,C show the population of Fluo-4-positive cells and cell viability after short-circuiting using HEPES-saline or HEPES-sucrose, respectively. Short-circuiting using HEPES-saline containing 10 mM Ca^2+^ resulted in a slight increase in the population of Fluo-4-positive cells relative to the control. Cell viability was decreased in both calcium ion concentrations. In contrast, short-circuiting using HEPES-sucrose resulted in a significant increase in the population of Fluo-4-positive cells relative to the control. A higher population of Fluo-4-positive cells was observed when using HEPES-sucrose containing 10 mM Ca^2+^ than in the absence of calcium ions. In addition, short-circuiting with and without calcium ions decreased cell viability. Remarkable cell death was observed in the presence of calcium ions.

As shown in Figure 2(A-2),B, short-circuiting using HEPES-saline without calcium ions did not increase in the population of Fluo-4-positive cells. Figure 2(D-1) shows typical flow cytometry histograms corresponding to Figure 2(A-1–A-3). Short-circuiting using HEPES-saline increased Fluo-4 fluorescence intensity compared with the untreated control. The presence of calcium ions showed a more significant increase in fluorescence intensity. Figure 2(D-2) shows the relative median Fluo-4 fluorescence intensity. The relative median Fluo-4 fluorescence intensity compared with the untreated control was obtained by dividing the median Fluo-4 fluorescence intensity by that of the untreated control using these histograms. Short-circuiting using HEPES-saline showed a statistically significant Fluo-4 fluorescence increase. The presence of calcium ions increased relative Fluo-4 fluorescence intensity.

### 3.2. YO-PRO-1 Uptake Assay

Figure 3 shows the results of permeabilization of YO-PRO-1 molecules measured by flow cytometry. Both transient permeabilization of YO-PRO-1 molecules and necrotic cell death can increase YO-PRO-1 fluorescence intensity; therefore, 7-AAD was added to stain dead cells 40 min after short-circuiting. Figure 3A shows representative flow cytometry density plots. In the case of HEPES-saline, as shown in Figure 3(A-2), short-circuiting resulted in a slight increase in the population of YO-PRO-1-positive cells relative to the untreated control (Figure 3(A-1)). Short-circuiting using RPMI-1640 showed a remarkable increase in the population of YO-PRO-1-positive cells (Figure 3(A-3)). As shown in Figure 3(A-4), short-circuiting using HEPES-sucrose resulted in the most significant increase in YO-PRO-1-positive cells; however, it increased the population of dead cells. Figure 3B shows the percentage of YO-PRO-1-positive viable cells and cell viability one hour after short-circuiting. The population of YO-PRO-1-positive viable cells was significantly increased after short-circuiting using RPMI-1640 and HEPES-sucrose. In the cases of HEPES-saline and RPMI-1640, no significant decrease in cell viability was observed, and more than 90% of the cells were viable in each condition. Short-circuiting using RPMI-1640 significantly increased the population of YO-PRO-1-positive viable cells compared with HEPES-saline. Short-circuiting using HEPES-sucrose also significantly increased the population of YO-PRO-1-positive viable cells; however, a significant decrease in cell viability was observed.

### 3.3. Measurement of the Loss of Intracellular Molecules

Figure 4 shows the results of monitoring the extracellular release of membrane-impermeable molecules. Figure 4A shows typical flow cytometry histograms after short-circuiting using different electroporation media. The histograms are representative of three independent experiments. Short-circuiting using HEPES-saline and RPMI-1640 did not result in a significant change in calcein fluorescence intensity. In the case of HEPES-sucrose, in contrast, the population of cells indicating a decrease in calcein fluorescence intensity was remarkably increased. Figure 4B shows the percentage of calcein-leaked cells after short-circuiting, as determined by flow cytometry. A statistically significant increase in the population of calcein-leaked cells was observed after short-circuiting using HEPES-sucrose.

### 3.4. The Effect of Endocytosis Inhibition on YO-PRO-1 Uptake

Figure 3 showed that the short-circuiting using RPMI-1640 enhanced YO-PRO-1 uptake compared to HEPES-saline, although the electric conductivities of these solutions and cell viability are almost the same. However, short-circuiting using RPMI-1640 did not show a significant extracellular release of calcein molecules, as shown in Figure 4. Therefore, we considered endocytic pathways as different YO-PRO-1 uptake pathways from membrane permeabilization. Figure 5 showed the effect of endocytosis inhibition on YO-PRO-1 uptake after short-circuiting using RPMI-1640. Figure 5A shows the effect of the pretreatment of cells with an endocytosis inhibitor on YO-PRO-1 uptake. Here, Pitstop 2-100, a clathrin inhibitor, was used. Flow cytometry assays were performed, as shown in Figure 3. No remarkable decrease in cell viability after short-circuiting was observed in each endocytosis-inhibitor concentration. Therefore, pretreatment of cells with Pitstop 2-100 did not affect cell health. However, the pretreatment significantly decreased the population of YO-PRO-1-positive viable cells. Figure 5B shows the temperature dependence of YO-PRO-1 uptake after short-circuiting. As almost all endocytic pathways are metabolic energy-dependent processes, the influence of incubation temperature on YO-PRO-1 uptake was examined. Incubation at 4 °C significantly decreased the population of YO-PRO-1-positive viable cells compared with incubation at 37 °C.

## 4. Discussion

This study aimed to investigate the transient membrane permeabilization stimulated by electrical short-circuiting of an aqueous droplet in dielectric oil. Among the various experimental parameters, we mainly focused on the ion strengths of an electroporation medium. We evaluated the membrane permeabilization by three methods: the influx of calcium ions, YO-PRO-1 uptake, and calcein leakage.

Figure 2 shows the results of intracellular calcium ion measurement. Flow cytometry was performed after incubation with 7-AAD. Fluo-4 fluorescence intensity corresponds to cytosolic calcium ion concentration. As shown in Figure 2(A-5,A-6),C, the short-circuiting using HEPES-sucrose increased the population of Fluo-4-positive cells. Although the changes in the population of Fluo-4-positive cells were small when using HEPES-saline, the relative median Fluo-4 fluorescence intensity was increased, as shown in Figure 2D. Therefore, Figure 2 indicates that short-circuiting using any electroporation medium increased cytosolic calcium ion concentration.

An increase in Fluo-4 fluorescence intensity was observed when using HEPES-saline and HEPES-sucrose containing 10 mM Ca^2+^ than in the absence of calcium ions. This result suggests that the short-circuiting stimulated membrane permeabilization, resulting in the influx of extracellular calcium ions. In addition, by comparing HEPES-saline and HEPES-sucrose, HEPES-sucrose containing 10 mM Ca^2+^ showed the highest population of Fluo-4-positive cells. This result indicates that an electroporation medium with low ion strength enhanced the membrane permeabilization.

As shown Figure 2, the short-circuiting using both HEPES-saline and HEPES-sucrose in the absence of calcium ions also increased cytosolic calcium ion concentration. This could be attributed to calcium leakage from endoplasmic reticulum (ER) stores. In addition, the short-circuiting using HEPES-sucrose in the absence of calcium ions showed a remarkably high population of Fluo-4-positive cells. This result was attributed to a more intense electric field that enhanced ER membrane permeabilization when using the low-ion-strength medium.

In Figure 2(A-5), we can confirm the increase in the population of both 7-AAD-positive cells and Fluo-4-positive viable cells when using HEPES-sucrose in the absence of calcium ions. Moreover, 7-AAD was added 20 min after the short-circuiting; therefore, an increase in 7-AAD fluorescent intensity indicates a failure of membrane repair, resulting in necrotic cell death. This result suggests that the short-circuiting using HEPES-sucrose in the absence of calcium ions stimulated both transient and irreversible membrane pore formation. Furthermore, Figure 3(A-4) also showed remarkable cell death. In measuring intracellular calcium ions, dead cells were stained with 7-AAD 20 min after electroporation. Meanwhile, 7-AAD was added 40 min after electroporation in the YO-PRO-1 uptake assay. The difference in the population of 7-AAD-positive cells between Figure 2(A-5) and Figure 3(A-4) could be attributed to the timing of dead cell staining. In Figure 2(A-6), in contrast, most cells are dead after short-circuiting using HEPES-sucrose containing 10 mM Ca^2+^. As previously reported, electroporation has been used in combination with calcium ions, and when delivered in excess concentrations can induce rapid cell death [27]. Therefore, the remarkable cell death observed in Figure 2(A-6) is attributed to irreversible electroporation and calcium-induced cell death.

Figure 3 shows the results of YO-PRO-1 uptake assays. We have already published YO-PRO-1 uptake stimulated by electric short-circuiting [33,35]; however, the effect of electroporation medium on YO-PRO-1 uptake was not investigated. YO-PRO-1 fluorophores are impermeant molecules that bind to nucleic acids and show significant fluorescence enhancement. Therefore, an increase in YO-PRO-1 fluorescence intensity indicates the uptake of YO-PRO-1 into cells. The short-circuiting using HEPES-saline and RPMI-1640 stimulated YO-PRO-1 uptake without inducing cell death. In the case of HEPES-saline, a slight change in the population of YO-PRO-1-positive viable cells corresponds to that of the influx in calcium ions, as shown in Figure 2. In contrast, the short-circuiting using HEPES-sucrose remarkably increased the population of YO-PRO-1-positive viable cells and dead cells. This result also agreed with the influx of calcium ions. By comparing HEPES-saline and RPMI-1640, the short-circuiting using RPMI-1640 enhanced YO-PRO-1 uptake more than HEPES-saline, although the electric conductivities of these solutions and cell viability are almost the same.

We evaluated the extracellular release of cell-impermeable molecules to investigate the inconsistency. Figure 4 shows the results of monitoring the extracellular release of membrane-impermeable calcein molecules. The short-circuiting using HEPES-sucrose decreased the calcein fluorescence intensity, indicating the extracellular release of calcein molecules. This result is consistent with the influx of calcium ions and YO-PRO-1 uptake. Therefore, the short-circuiting using an electroporation medium with low electric conductivity enhanced both transient and irreversible membrane pore formations. In contrast, the short-circuiting using HEPES-saline did not show a significant extracellular release of calcein molecules, although the short-circuiting stimulated the influx of calcium ions and YO-PRO-1 uptake. The size of calcein (MW = 623) is quite different to that of calcium; however, YO-PRO-1 (MW = 629) is comparable with calcein. In addition, the short-circuiting using RPMI-1640 also did not show a significant extracellular release of calcein molecules. As shown in Figure 3, the short-circuiting using RPMI-1640 enhanced YO-PRO-1 uptake more than using HEPES-saline. Therefore, we considered different YO-PRO-1 uptake pathways from membrane permeabilization.

Figure 5 shows the effect of endocytosis inhibition on YO-PRO-1 uptake. Endocytosis has recently been considered as a possible pathway for exogenous gene expression by conventional gene electrotransfection [37,38,39]. We also have reported that short-circuiting via an aqueous droplet stimulated endocytosis of plasmid DNA molecules [35]; however, the contribution of endocytosis to the delivery of small molecules remains to be investigated. Figure 5A shows the effect of pretreatment of cells with endocytosis inhibitor Pitstop 2-100 on YO-PRO-1 uptake. The pretreatment significantly decreased the population of YO-PRO-1-positive viable cells. Since Pitstop 2-100 inhibits clathrin function [36], Figure 5A suggests that clathrin-mediated endocytosis could be a possible molecular pathway for YO-PRO-1 uptake investigated in this study. However, the difference between 10 and 20 μM Pitstop 2-100 in the population of YO-PRO-1-positive viable cells was not statistically significant; therefore, the concentration dependency on YO-PRO-1 uptake was not confirmed. A much higher concentration of Pitstop 2-100 could be responsible for this result. Our previous study investigated the influence of the pretreatment of Jurkat cells with 5, 10, and 20 μM Pitstop 2-100 on gene transfection by short-circuiting [35]. As a result, pretreatment with 10 and 20 μM Pitstop 2-100 decreased the transfection efficiency; however, 5 μM did not. The result displayed in Figure 5A is consistent with our previous result. In addition, incubation at 4 °C also suppressed YO-PRO-1 uptake, as shown in Figure 5B. Since endocytic pathways are metabolic energy-dependent processes, this result supports that endocytosis contributed to YO-PRO-1 uptake. Therefore, these results suggested that clathrin-mediated endocytosis contributed to YO-PRO-1 uptake when using RPMI-1640 as an electroporation medium. However, the other endocytic pathways, caveolae-mediated endocytosis, macropinocytosis, and phagocytosis [40,41], remain to be elucidated. Further experiments with different inhibitors for specific pathways will be investigated in the future.

As many previous reports have shown, YO-PRO-1 uptake via pores and endocytotic processes can occur during and following electrical stimulation. To explore this phenomenon, Antov et al. reported that nonpermeabilizing pulsed trains of low electric fields (LEF) in the range of 2.5–20 V/cm stimulated uptake of FITC dextran, BSA-FITC, Lucifer yellow, and PI via endocytotic pathways independent of their molecular weight and charge [42,43]. In addition, Ben-Dov et al. demonstrated that electrochemical production of protons at the anode interface is responsible for inducing the uptake of macromolecules [44]. Recently, Yadegari-Dehkordi et al. investigated that low-voltage and high-frequency electric fields stimulate different endocytosis pathways in MCF-7 cells to enhance uptake of bleomycin [45]. YO-PRO-1 uptake stimulated by the short-circuiting shown in Figure 3 and Figure 5 could be attributed to pores and endocytotic processes. The short-circuiting could induce a local change in pH of the droplet; however, further investigation is required to elucidate the mechanism.

We evaluated the effect of an electroporation medium on the membrane permeabilization by three methods, influx of calcium ions, YO-PRO-1 uptake, and calcein leakage. As a result, an electroporation medium with low electric conductivity stimulated more transient and irreversible membrane pore formation than high electric conductivity. Electroporation depends on the polarization of cells induced by a pulsed electric field and transient charge accumulation on the cell membrane. When transmembrane potential reaches a threshold, membrane permeability is increased due to transient membrane pore formation. In addition to various parameters of a pulsed electric field, the polarization depends on the permittivity and conductivity of both the cells and the electroporation medium. Therefore, the effects of electric conductivity of an electroporation medium on transient membrane permeabilization have been focused on for decades [46,47,48,49,50,51,52]. Rols and Teissie reported that membrane permeabilization of Chinese hamster ovary cells increased with increasing ionic strength of the medium [46]. In contrast, Ruzgys et al. pointed out that several published studies show that the transient permeabilization is inversely proportional to the electric conductivity of the medium. They also suggested that the cell deformation process induced by the higher conductivity decreases transmembrane potential, resulting in transient pores or electroporated areas becoming smaller [50]. Novickij et al. also demonstrated that the lower-conductivity medium enhances the permeabilization during microsecond range pulses; in contrast, the lower conductivity will reduce the permeabilization for sub-microsecond-range pulses [52]. A similar phenomenon possibly occurs during the short-circuiting; however, further experiments will be required to elucidate the detailed mechanism of the effect of extracellular medium on the delivery of small molecules.

We investigated YO-PRO-1 uptake and calcein leakage stimulated by short-circuiting. Although these fluorescent dyes are similar in size, the electrical charges of these molecules are different (YO-PRO-1: +2, calcein: −4). Sözer et al. investigated the influx of PI and YO-PRO-1 (cations) and calcein (anion), and the efflux of calcein [26]. They showed that the influx of these cations was much more significant than the influx of calcein; in contrast, the efflux of calcein was equivalent to the influx of PI and YO-PRO-1. These relative transport rates are correlated not with molecular size but rather with molecular charge polarity. The comparison investigated in this study should consider these characteristics; however, this consideration remains to be elucidated.

In conclusion, the electric conductivity of the electroporation medium affected the delivery of small molecules stimulated by short-circuiting via an aqueous droplet containing Jurkat cells. Low-conductivity medium-enhanced small molecule transfer was demonstrated by three methodological approaches. Clathrin-mediated endocytosis contributed to YO-PRO-1 uptake when RPMI-1640 was used as an electroporation medium.

## Data Availability

Not applicable.
